# Comprehensive analysis of 65 patients with Castleman disease in a single center in China

**DOI:** 10.1038/s41598-022-12797-y

**Published:** 2022-05-24

**Authors:** Xi-Qian Wang, Nian-Nian Zhong, Qi Sun, Si-Chen Yan, Guang-Cai Xu, Yong-Gong Wang, Li-Wei Peng, Bing Liu, Lin-Lin Bu

**Affiliations:** 1 Department of Oral & Maxillofacial Surgery, Henan Provincial People’s Hospital, Zhengzhou University People’s Hospital, Henan University People’s Hospital, Zhengzhou, 450003 Henan China; 2 The State Key Laboratory Breeding Base of Basic Science of Stomatology (Hubei-MOST) & Key Laboratory of Oral Biomedicine Ministry of Education, School & Hospital of Stomatology, Wuhan University, Wuhan, 430079 Hubei China; 3 Department of Pathology, Henan Provincial People’s Hospital, Zhengzhou University People’s Hospital, Henan University People’s Hospital, Zhengzhou, 450003 Henan China; 4 Department of Oral & Maxillofacial Head Neck Oncology, School & Hospital of Stomatology, Wuhan University, Wuhan, 430079 Hubei China

**Keywords:** Cancer, Surgical oncology

## Abstract

This study aimed to investigate the epidemiologic, clinical, pathological characteristics, and treatment of patients with Castleman disease (CD) in a single center in China. We retrospectively analyzed the data of 65 Chinese CD patients, divided into unicentric CD (UCD) and multicentric CD (MCD) groups, and also microscopic subtypes as hypervascular (HV), plasmacytic (PC) and Mixed. Based on whether HHV-8 infection existed, MCD was subdivided into HHV-8-associated MCD and idiopathic Castleman disease (iMCD). Detailed epidemiologic, clinicopathological, and treatment data were analyzed and discussed. Of total 65 patients (UCD 33, MCD 32), HV (81.8%) accounted for the most of UCD and total. More females in UCD (60.6%) and more males in MCD (65.6%) were observed. CD occurred in all age groups, most commonly in 40–49 years. The mean age of onset of total was 38.5 years with PC higher than HV (45.5 vs. 35.1 years, P = 0.0413). The median diagnosis delay of MCD was longer than that of UCD (3.00 vs. 1.25 months, P = 0.0436). Abdomen (39.4%) and neck (30.3%) were the most-seen locations of lymphadenopathy in UCD, with neck (65.6%) being predominant in MCD. Mean major diameter of specimens of UCD was greater than MCD (6.4 vs. 3.1 cm, P < 0.0001). These results provided the featured and detailed profile of Castleman disease in Henan province in China with a considerable number of cases, which presented distinct evidence with other studies.

## Introduction

Castleman disease (CD), which was originally reported in 1950s by Dr. Benjamin Castleman as localized mediastinal lymph node enlargement^1^, is a heterogeneous group of lymphoproliferative disorders usually associated with histologic presentations of angiofollicular lymph-node hyperplasia with capillary proliferation, hyperplasia of lymphoid follicles, and cellular infiltration of plasma cells^2^. Based on its histopathological features of lymph nodes, CD can be classified into three subtypes under microscope: the hyaline vascular type (HV), the plasma cell type (PC) and the Mixed (or intermediate) type in between^3,4^. Not just with microscopic classification, CD is usually diagnosed by doctors according to how much the body is affected. In this way, it is divided into unicentric CD (UCD) and multicentric CD (MCD) regarding the clinical presentation—a UCD patient has a single enlarged lymph node or region of lymph nodes and a MCD one involves multiple lymph node stations, which is subdivided into human herpes virus-8 (HHV-8)-associated MCD and idiopathic MCD (iMCD)^5–7^. CD acts similarly to lymphomas that MCD could develop into and the treatment of MCD often refers to that of lymphoma too, which is why CD is included in the American Cancer Society’s cancer information.

The causes, clinical features, and outcomes of CD vary from one subtype to another. A neoplastic follicular dendritic cell population in all probability contributes to the cause of UCD, whereas HHV-8 infection as well as a monoclonal plasma cell population prove to be the etiological drivers of MCD (iMCD still remains less clear)^8^. HHV-8 is also known as Kaposi sarcoma herpesvirus (KSHV). HHV-8 positive MCD often occurs in cases caused by HIV infection or other immune deficiency^9^. The diagnosis of CD includes clinical examinations (specific clinical signs and complications), imaging study (systemic imaging with PET/computed tomography), laboratory investigations, pathological evaluation with immunostaining as well as molecular virology. Besides the other more common causes of lymphadenopathy, lymphoma, autoimmune connective disorders, infectious diseases including chronic active Epstein–Barr (EB) virus infection, and IgG4-related diseases are usually for the differential considerations of CD diagnosis^2,10,11^. The most common location for UCD is mediastinum, whereas less common sites include neck, axilla, abdomen, and pelvis. In comparison with UCD, MCD patients are characterized by constitutional symptoms (including fever, weight loss, fatigue, night sweats, and nerve damage that can cause weakness and numbness), organomegaly and more aggressive clinical course with the potential for malignant transformation^10^. Surgical removal is strongly recommended for UCD regardless of its microscopic subtype and is usually curative, but the treatment decision for MCD needs more careful consideration hinging on its specific subclassification^5,9^.

Despite all this, the very nature of CD still remains unclear enough, especially the little published information about incidence from China. As a rare entity, CD received its first ICD-10 diagnosis code in October 2016, but overall standard diagnostic criteria are still needed to be improved, especially iMCD^12^. In this study, we retrospectively analyzed 65 patients in Henan Provincial People’s Hospital in central China for the better understanding of this disease. These data covered a full spectrum of CD patients, by which more thorough features were evaluated, such as the epidemiologic, geographic characteristics and the admission departments distribution. A single-center data specificity with a fair number of patients was demonstrated. Furthermore, distinctively, a collection of comparative analyses were investigated and discussed. In general, more evidence and insights were given for further investigations of this disease.

## Methods

### Study design and subjects

This was a retrospective and observational study from a single center in real world. All the 65 patients were searched in the pathology database of Henan Provincial People’s Hospital with the definitive histopathological CD diagnosis between November 2011 and November 2020. Henan Provincial People’s Hospital was one of the largest general hospitals in Henan Province in central China where all 65 patients with CD in this study accepted the surgical treatment. Eighteen of them also were treated with chemotherapy and one was treated with radiotherapy. All the detailed epidemiologic, clinical, radiological, pathological, treatment, and follow-up data available were collected and analyzed. The CD classifications (UCD/MCD, HV/PC/Mixed) were determined by clinical and pathological evaluation respectively in accordance with generally accepted guidelines^4,5,13^. This study was reviewed and approved by the Medical Ethics Committee of Henan Provincial People’s Hospital and conducted conforming to the ethical requirements of biomedical research promulgated by the international and national governments.

### Statistical analysis

All the epidemiologic, clinical, and pathological characteristics and treatment data were collated and tabulated using Microsoft Excel. Statistical analyses were performed using IBM SPSS Statistics 26.0 (Armonk, NY, USA) and GraphPad Prism 9 (La Jolla, CA, USA). Descriptive statistics were analyzed for the variables of interest. Shapiro–Wilk tests were used to check the normality assumption. Quantitative variables were described using number (%) and mean ± SD (min–max) or median. The homogeneity of variances between two groups were assessed by Levene's test and the differences between two groups were compared using t-test or Mann–Whitney U test. A P value < 0.05 was considered statistically significant.

### Ethical approval

This study was conducted according to the guidelines of the Declaration of Helsinki, approved by the Medical Ethics Committee of Henan Provincial People’s Hospital, and performed in accordance with the ethical requirements of biomedical research promulgated by the international and national governments.

### Consent to participate/consent to publish

The need for informed consent was waived by the committee.

## Results

### Epidemiologic characteristics

**Figure 1:**
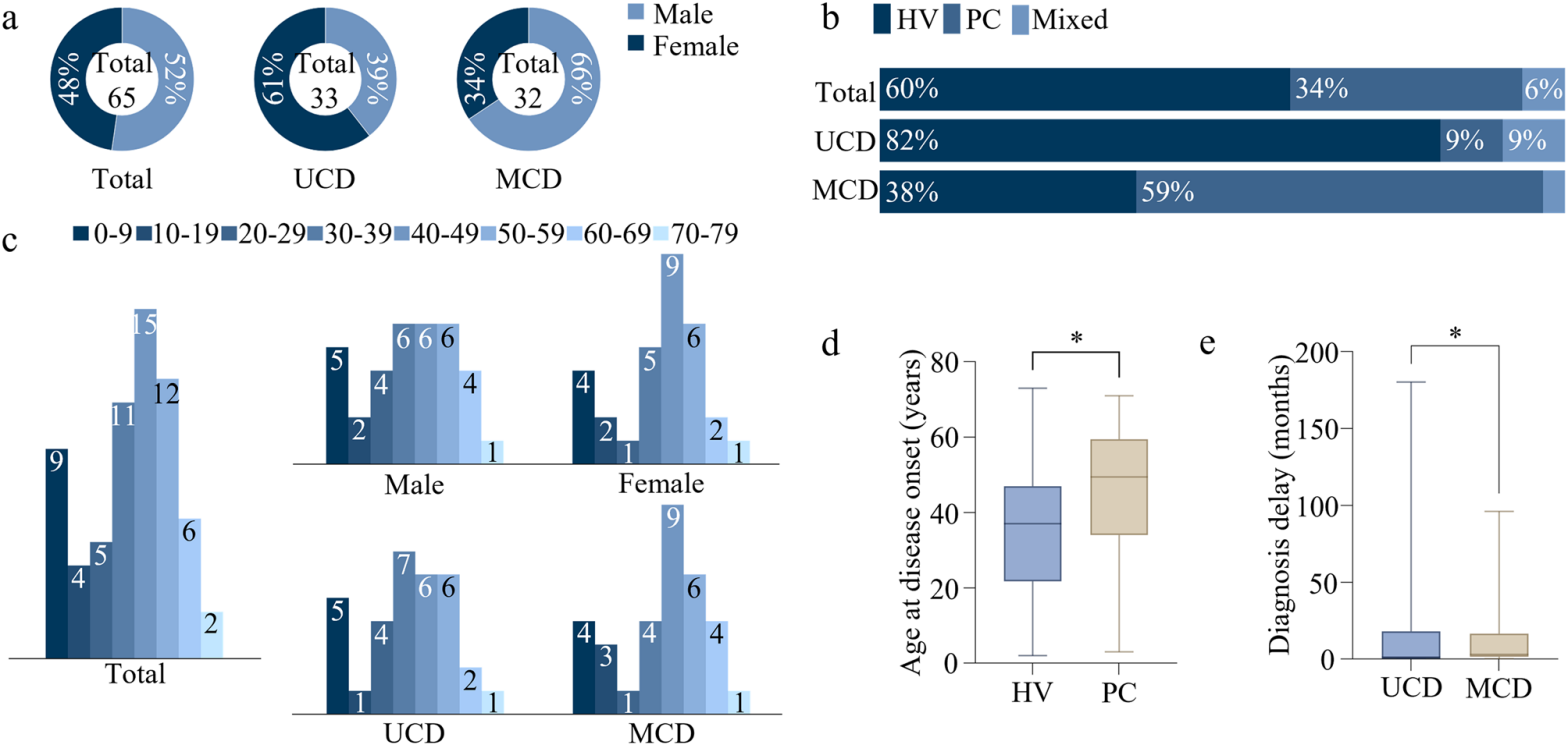
Epidemiologic characteristics. (**a**) Proportions of male and female in total, UCD and MCD patients; (**b**) Proportions of three microscopic subtypes of CD in total, UCD and MCD patients; (**c**) Distribution of CD patients in each age group; (**d**) The ages at disease onset (years) of HV and PC patients. *P = 0.0413 as determined by two-tailed Student’s t test. (**e**) The diagnosis delays (months) of UCD and MCD patients. *P = 0.0436 as determined by two-tailed Mann–Whitney U test.

A total data of 65 patients with biopsy-proven CD diagnosis were included in this study, among which 50.8% (33/65) were classified into UCD and 49.2% (32/65) into MCD. Male patients accounted for 52.3% (34/65) with females two less (47.7%, 31/65) (Fig. 1a, Supplementary Table S1). However, it showed a small different trend in the subtypes that the female patients made up 60.6% (20/33) of UCD but male 65.6% (21/32) of MCD. In three histopathological subtypes, HV patients were the biggest subgroup (60.0%, 39/65), PC second (33.8%, 22/65), and Mixed type the least (6.2%, 4/65) (Fig. 1b, Supplementary Table S1). Among 33 patients presented with UCD, there were 27 with HV subtype (81.8%), 3 with PC (9.1%), and 3 with Mixed subtype (9.1%). Instead, among 32 patients with MCD, there were 19 with PC (59.4%), 12 with HV (37.5%), only 1 with Mixed type (3.1%).

Ages at disease onset were collected in 64 patients, in which UCD and MCD account for a precise half respectively. These ages were normally distributed, and thus showed using mean ± SD (min–max). The mean age at disease onset (years) of total patients was 38.5 ± 18.9 (2–73); that of 38 patients of HV subgroup was 35.1 ± 18.5 (2–73), and 22 patients of PC subgroup 45.5 ± 18.6 (3–71), significantly higher than HV subgroup (P = 0.0413) (Fig. 1d, Supplementary Table S1). The mean ages at disease onset of UCD and MCD were 36.1 ± 18.0 (2–73) and 40.9 ± 19.9 (3–71), respectively (Supplementary Table S1). As age distribution among 64 patients was showed in Fig. 1c, the percentage of patients in each age group was as follows: 0–9 (14.1%), 10–19 (6.3%), 20–29 (7.8%), 30–39 (17.2%), 40–49 (23.4%), 50–59 (18.8%), 60–69 (9.4%) and 70–79 (3.1%). The most frequently observed age groups among study were 40–49 years, and patients aged 30–39, 50–59 and 0–9 were also well represented (Fig. 1c).

The diagnosis delays (months) of these 64 patients did not conform to a normal distribution, so was represented using the median, which was 2.50. The medians of diagnosis delays of 32 UCD patients and 32 MCD patients were 1.25 and 3.00, respectively; furthermore, the medians of 38 patients with HV variant and 22 patients with PC variant were 2.00 and 2.50 (Supplementary Table S1). A significant difference was observed between diagnosis delays of UCD and MCD (P = 0.0436) (Fig. 1e).

All 64 patients with the data of birthplaces were analyzed, one of which came from Wuhan (Hubei Province) and the other from Meishan (Sichuan Province). The rest 62 patients were all from Henan Province. Supplementary Fig. S1 illustrated the places of origin of patients from Henan Province.

For the admission departments of 65 patients, Dept. of Gastrointestinal Surgery (11) had the largest number of visits, then Dept. of Pediatric Surgery (9), Dept. of Oral and Maxillofacial Surgery (8), Dept. of Hepatobiliary Pancreatic Surgery (7), Dept. of Thoracic Surgery (6), Dept. of Hematology (5), Dept. of General Surgery (4), Dept. of Thyroid Surgery (3), Dept. of Urology Surgery (3) and Dept. of Gastroenterology (3) (Supplementary Fig. S2).

### Clinical and radiological findings

**Figure 2:**
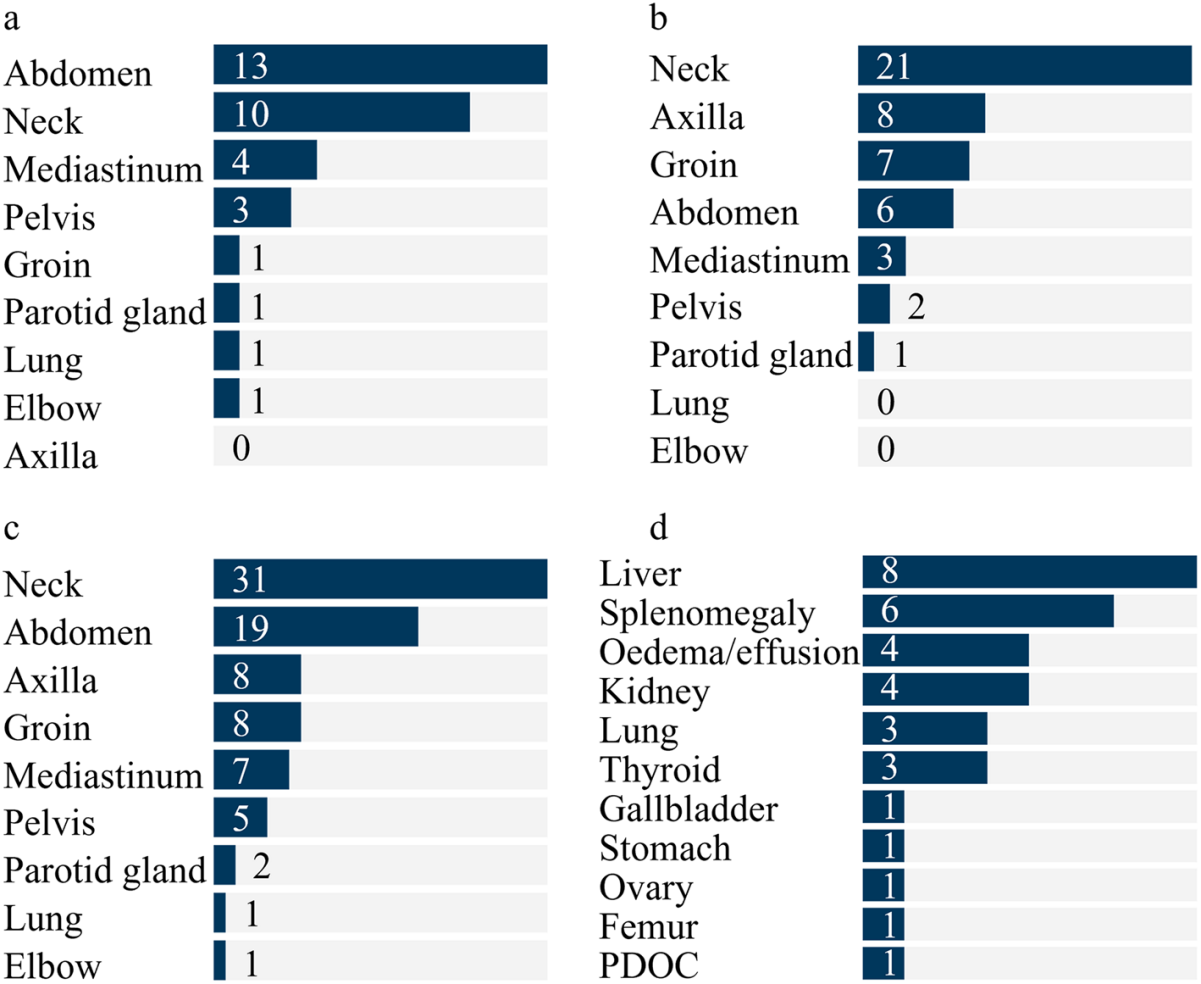
Clinical findings. (**a**) Locations of lymphadenopathy of 33 UCD patients; (**b**) Locations of lymphadenopathy of 32 MCD patients; (**c**) Locations of lymphadenopathy of total 65 patients; (**d**) Comorbidities in 65 patients with CD in this study.

Locations of lymphadenopathy in the body of 65 patients in this study were observed in neck (31, 47.7%), abdomen (19, 29.2%), axilla (8, 12.3%), groin (8, 12.3%), mediastinum (7, 10.8%), pelvis (7.7%), parotid gland (2, 3.1%), lung (1, 1.5%) and elbow (1, 1.5%) (Fig. 2a–c, Supplementary Table S2). Furthermore, the most frequent location in MCD patients was neck (21, 65.6%), while abdomen (13, 39.4%) and neck (10, 30.3%) were the most frequent locations in UCD patients. Supplementary Fig. S3 represented the enlarged lymph nodes on multiple planes of CT findings in neck, mediastinum, axilla, abdomen, groin and pelvis of 6 patients with CD.

Clinical signs and symptoms presented in 10 patients (15.4%) of a total 65, all of which were with MCD. All the 10 patients had the sign or symptom of fever (31.3%), 2 had cough (6.3%) and 1 had marasmus (3.1%) (Supplementary Table S2). 19 patients (29.2%) were with other comorbidities, the organs/diseases of which were in order of descending frequency as follows: liver (12.3%), splenomegaly (9.2%), oedema/effusion (6.2%), kidney (6.2%), lung (4.6%), thyroid (4.6%), gallbladder (1.5%), stomach (1.5%), ovary (1.5%), femur (1.5%), paroxysmal disturbance of consciousness (1.5%) (Fig. 2d, Supplementary Table S2). Comorbidities occurred in 9 patients (27.3%) of 33 UCD patients and 10 (31.3%) of 32 MCD patients.

### Pathological examinations

A histopathological examination for CD was required for a definitive diagnosis. The biopsy specimens were sent to the Department of Pathology for a routine HE stain. Supplementary Fig. S4a-b showed representative HE-stained tissues of an enlarged lymph node under microscope of an HV and a PC variant respectively. Usually, further pathological tests were required for a confirmed diagnosis. The disease-oriented doctors’ choices of pathological indexes, including immunohistochemistry (IHC) and molecular pathology, for 65 patients with CD before diagnosed were summarized (Supplementary Fig. S5). Indexes chosen more than 40 times were Ki67, CD20, CD3, CD21, Pax-5, CD23, CD10, and EBER. Supplementary Fig. S4c–j represented the characteristic IHC staining of CD3, CD4, CD8, CD20, CD21, CD34, Ki67 (HV), Ki67 (PC), on which the diagnoses were made based. 15 patients were examined with HHV-8 using polymerase chain reaction (PCR) or IHC staining, including 8 patients with UCD and 7 with MCD (Supplementary Table S3). Only 1 case (14.3%) with MCD was HHV-8 positive.

**Figure 3:**
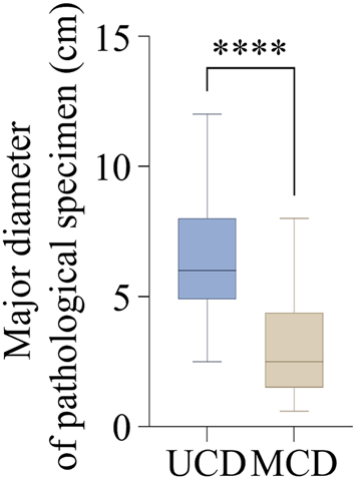
Pathological findings. The major diameters of pathological specimens (cm) of UCD and MCD patients. ****P < 0.0001 as determined by two-tailed Student’s t test.

The major diameter (cm) of biopsy specimens from 65 patients with CD were analyzed, which conformed the normality assumption. The mean major diameters of specimens of total 65 patients, 33 patients with UCD, 32 patients with MCD were 4.7 ± 2.6 (0.6–12.0), 6.4 ± 2.2 (2.5–12.0) and 3.1 ± 1.9 (0.6–8.0), respectively (Supplementary Table S3). The mean major diameter of specimens of UCD patients was larger than that of MCD patients with a statistical significance (P < 0.0001) (Fig. 3).

### Treatment outcomes

All 65 patients in our study with CD, including 33 (50.8%) patients with UCD and 32 (49.2%) patients with MCD, accepted surgical treatment (Table 1). 2 (6.1%) of 33 patients with UCD received chemotherapy after the surgery. Since there was no unified standard for treatment of MCD, it varied among patients. 1 (3.1%) of 32 patients with MCD received radiotherapy, while 16 (50.0%) of them were treated with chemotherapy. Detailed chemotherapy regimens were showed in Supplementary Table S4. By the end of December of 2021, the median follow-up time (months) of all 65 patients was 66.0 (Table 1). The recurrence of CD occurred in 2 (6.3%) patients with MCD (both accepted no chemotherapy) and 3 (9.4%) patients with MCD died (two died of CD progression and one died of prostate cancer). Favorable prognoses were observed in all UCD patients.

**Table 1:** Treatment choices and treatment outcomes.

	UCD	MCD	Total
**Treatment, n (%)**	33 (50.8%)	32 (49.2%)	65
SE	31 (93.9%)	15 (46.9%)	46 (70.8%)
SE + RT	0	1 (3.1%)	1 (1.5%)
SE + CT	2 (6.1%)	16 (50.0%)	18 (27.7%)
**Follow-up time (months), median, n (%)**	53.0, 33 (50.8%)	79.5, 32 (49.2%)	66.0, 65
**Recurrence, n (%)**	0	2 (6.3%)	2 (3.1%)
**Survival, n (%)**	33 (100%)	29 (90.6%)	62 (95.4%)

*SE* surgical excision, *RT* radiotherapy, *CT* chemotherapy.

## Discussion and perspective

Although it has been more than 60 years since the first time CD was reported and consensus on the diagnostic criteria has been developed, due to its rarity and clinical heterogeneity, a further understanding still need to be deepened in the diagnosis and management for specialists^9,14^. Here we comprehensively studied the data between patients with UCD and MCD in Henan Provincial People’s Hospital in central China to provide more information focusing on its epidemiologic, clinical, and pathological characteristics. In this study, some of our data were similar to others previously reported but some were different. This could be caused by various factors, including unbalanced economic levels in regions/countries, differentiated patients’ awareness of seeking medical care, diverse modes of general hospitals and specialized hospitals, differences among researchers’ departments of work, dissimilarity between single-center and multi-center studies, distinct nations or races, etc.

In our study in Henan Provincial People’s Hospital and the previous study in the First Affiliated Hospital of Zhengzhou University, patients with UCD and MCD tended to be half to half (50.8%/49.2%, 49.3%/50.7%); similarly, UCD made up of 47.6% (69/145) in a series from Beijing (China)^15,16^. However, UCD accounted for 79.7% (47/59) in a study in the Henan Cancer Hospital; in a multicenter study of 185 patients in China, UCD accounted for 65.4%; likewise, in a systematic review published in 2012, UCD comprised 68% of 404 cases; this percentage in USA was described as 75%^9,17–19^. The MCD/UCD ratio seemed to be higher in Henan Province than in other regions in China or in other countries. For the pathological variants, HV subgroups were the most in three subtypes in UCD patients and in total in three studies in Henan over the approximate timespan^15,17^. This was also consistent with other reports from Nebraska (USA), Beijing (China) and Paris (France)^20–22^. Whereas MCD included HV subgroup as the most (47.22%) in the study from the First Affiliated Hospital of Zhengzhou University, which was contrary to our study and a recent review report that PC was the most in MCD^9^. In our study and the study from Henan Cancer Hospital, the sex ratio of male to female was approximately 1:0.9 (52.3%/47.7%, 52.5%/47.5%), and that of the First Affiliated Hospital of Zhengzhou University was about 1:1.1 (47.9%/52.1%), showing no significance in gender difference^15,17^. The sex predominance varied in two clinical types: UCD showed a mild female preponderance in our study (60.6%), as well as other three reports from Zhengzhou (China) (53.9%), Madrid (Spain) (75%) and a systematic review (60%); MCD showed a male predominance in our study (65.6%)^15,18,23^. A study in Japan showed a similar sex ratio (male: 59.1%) in MCD as our study, as well as an epidemiologic review in 2018^24,25^. All ages groups were affected by CD with a high incidence between 30 and 59 years old (Fig. 1c). The 2 types of CD differed in ages at disease onset, with a mean age of 35.1 for HV variant and 45.5 for PC variant, and the mean age of onset (years) of total in this study was 38.5. And also, the mean age of MCD (40.9) was higher than that of UCD (36.1) (Supplementary Table S1). This was also close to previous studies in the First Affiliated Hospital of Zhengzhou University (40.3) and the Henan Cancer Hospital (40) (take consideration of the diagnosis delay), as well as other reports from domestic and overseas^9,15,17,19,25,26^. The diagnosis delay of MCD patients (3.00 months) in our study was longer than UCD patients (1.25 months). This may have been attributed to that patients with UCD developed clinical signs (enlarged lymph nodes) earlier than those with MCD. A French pediatric cohort study reported a similar trend of diagnosis delay between UCD (8.16 months) and MCD (5.16 years), which might be the reason that the subjects were in different age groups^27^. As a provincial hospital, it can be seen that the majority of our patients came from Henan Province but outside Zhengzhou (Supplementary Fig. S1).

Interestingly, different from the previous report that UCD occurred most in the mediastinum, the most common locations of enlarged lymph node in UCD patients in our study were abdomen and neck and in MCD patients was neck^5,28^. Nonetheless, our investigation agreed to the reports from the First Affiliated Hospital of Zhengzhou University^15^. Less common sites of CD also occurred in our study, such as the elbow and the parotid gland, which have been reported before^29,30^. Compared with UCD usually without obvious symptoms in chief complaint, MCD patients was prone to symptoms such as fever. The incidences of comorbidities between UCD and MCD were similar and at a low level (27.3% and 31.3%). Among the comorbidities, liver diseases and splenomegaly appeared more frequent in CD, which may have a relationship with immunity decrease. Besides, paraneoplastic pemphigus, autoimmune hemolytic anemia, Sjogren’s syndrome, myasthenia gravis, and psoriasis were also reported to be with CD^31^. Due to the various symptoms or locations of the disease, the admission departments were also widely distributed, and the department where patients were most likely to be treated was the Dept. of Gastrointestinal Surgery (Supplementary Fig. S2).

We showed all pathological examination indexes of 65 cases (including molecular pathological indexes) in Supplementary Fig. S5, which were used for the diagnosis and differential diagnosis of CD. Among them, the commonly used diagnosis indexes for lymphatic system diseases are as follows. CD20, CD79a, Pax-5, Kappa, Lambda, cyclin-D1 are often used as B cell markers; CD3, CD2, CD5, CD7, CD4, CD8, CD43 are commonly used for T cell labeling; CD10, Bcl-6 and CD38 can be used as germinal center indicators; FDCs, or follicular dendritic cells, are immunolabeled with CD21, CD23, CD35; and other lymphocyte markers as CD138, Bcl-2, Mum-1, CD34, CD30, CD163, CD15, TdT, LCA, etc.; Ki67 is a cell growth index, representing the cell growth rate in the cell cycle except for the G0 phase and serving as a powerful diagnostic tool in the evaluation of lymphoproliferative disorders^32^.What is also worth noticing is that the mean major diameter of pathological specimens (cm) of UCD patients exceeded MCD (Fig. 3), suggesting a possible correlation between the enlargement of lymph node and its low malignant potential. Only one (pathological subtype: PC) in fifteen patients in our study was tested HHV-8 positive, which was a very low proportion compared to reports in France and sub-Saharan Africa^22,33^. Other than pathological examinations, in CD diagnosis, surgical design, and postoperative examination, imaging examinations were also preferred and essential^34,35^.

Complete surgical resection is often curative and thus preferred as the first-line therapy for UCD, except unresectable UCD, of which symptomatic ones require rituximab with or without steroids, or anti-IL-6 therapies^4^. Progresses in the research and treatment of CD are largely coordinated by the Castleman Disease Collaborative Network (CDCN) (https://cdcn.org/). Previous literature reports are mostly based on the treatment experience of non-Hodgkin’s lymphoma. According to latest consensus on management of MCD, B cell-directed monoclonal antibody therapy (rituximab) is the preferred for HHV-8 positive MCD and the first IL-6 monoclonal antibody, siltuximab, which was approved by the United States of America (USA) Food and Drug Administration (FDA), or tocilizumb (if siltuximab is not available), is the preferred first-line therapy for iMCD^9,13,36,37^. Clinical trials towards siltuximab in iMCD patients were also actively performed to validate its long-term safety and activity as well as to find its predictive biomarkers of response^37–39^. However, currently, the anti-IL-6 therapy (siltuximab), is not widely applicable and does not benefit all patients with CD (nonresponders)^13,40^. Studies on alternative treatment approaches that can be adaptively used were also rigorously proceeded, such as the thalidomide-cyclophosphamide-prednisone (TCP) regimen, which proved to be an effective and safe treatment of newly diagnosed iMCD patients^41^. In this study over the past decade, CHOP was the main chemotherapy regimen chosen; thalidomide and rituximab treatment were also used (Supplementary Table S4). Recently, the consensus of the diagnosis and treatment of CD in China was released based on international consensus and experts experience in China, and soon after Sylvant® (siltuximab injection, for intravenous infusion) was approved by the National Medical Products Administration (NMPA) to treat adult patients with iMCD, which provided a helpful treatment option for patients suffering from this rare disease in China^42^. According to the consensus, the recommended first-line regimens for non-severe iMCD were as follows: (a) siltuximab ± prednisone; (b) TCP regimen; (c) R-CVP regimen (rituximab + cyclophosphamide + vincristine + prednisone); (d) rituximab ± prednisone. Recommended regimens for severe iMCD was the first-line combination of siltuximab and high-dose corticosteroids (such as methylprednisolone)^42^. Besides, recent studies focusing on the cellular and molecular mechanisms, genomic alterations of CD, and other biological agents also revealed candidate therapeutic targets and drugs for its treatment^43–48^.

The treatment of CD in a single department is generally summarized experience, plus the rarity characteristic and clinical heterogeneity it owns, so there are diverse perceptions of this disease among doctors from different departments. Therefore, we suggest that a multi-disciplinary team (MDT) for rare diseases, a working group made up of experts from departments of surgery, oncology, radiology, pathology, radiotherapy, etc., should be applied to propose the best treatment plan suitable for patients through a regular consultation. The treatment program is supposed to be implemented then by a combination of disciplines, and a forum of rare diseases should be recommended strongly, no matter online or in the real world, given the epidemic reality. Based on advancements in digital technologies and communication engineering, 5G + telemedicine is also recommended for the management of rare diseases, which will benefit the grassroots medical environment^49,50^. This kind of connection form among related departments, though weak linked, could enhance doctors’ cognition and encourage the diagnosis and management of rare diseases^51^.

Although our study is a good representation of a subset of patients with CD, there are some limitations. First, a notable limitation is the small subject size of CD patients so that we failed to make survival analyses and prognostic evaluations, though we compared our study with two other centers in the same area. Second, we did not obtain the laboratory test results to make a further comprehensive assessment for CD patients. Third, HHV-8 tests were not performed to every patient with MCD, so further and vital classifications for MCD subgroups was restricted.

In conclusion, we summarized these data from one single center as well as compared them with data from other centers. Some differences are revealed between our study and others; further investigations are needed to confirm these differences among regions, countries, and races. Our research provided evidence for more comprehensive analyses of CD in the future, to improve the understanding of this rare disease. Moreover, we propose that the MDT pattern for rare diseases should be applied into clinical practice.

## Supplementary Information

Supplementary Information.

## Data Availability

Data analyzed for this publication are available from the corresponding author on reasonable request.
