# Supplementary material for: Comprehensive analysis of 65 patients with Castleman disease in a single center in China
Source: Sci Rep. 2022 May 24;12:8694. doi: 10.1038/s41598-022-12797-y (PMC9130315; doi:10.1038/s41598-022-12797-y)
Supplement: Supplementary file 1 — Supplementary Information. [file 41598_2022_12797_MOESM1_ESM.pdf]

**Supplementary Information**

**Comprehensive analysis of 65 patients with Castleman disease in a single center in China**

Xi-Qian Wang[1,2,†], Nian-Nian Zhong[2,†], Qi Sun[3,†], Si-Chen Yan[1], Guang-Cai Xu[1], Yong-Gong Wang[1],

Li-Wei Peng[1,*], Bing Liu[2,4,*] and Lin-Lin Bu[2,4,*]

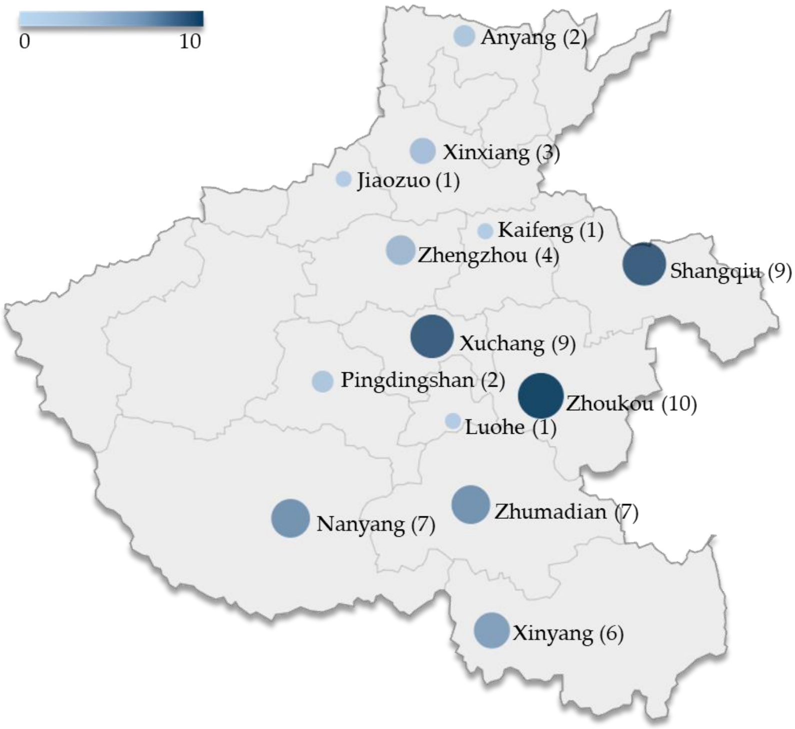


**Supplementary Figure S1. Birthplaces of patients.** 62 patients with CD from Henan Province in central China were distributed in 13 cities.

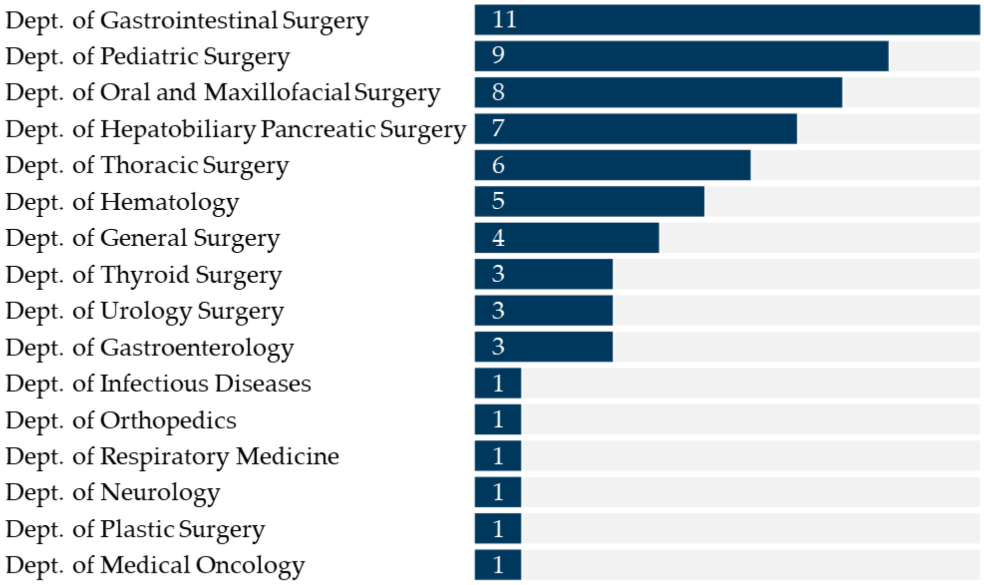


**Supplementary Figure S2. Admission departments.** Data of 65 patients with CD were collected from 16 departments in the hospital. Distribution of their source departments revealed the most frequent initial admission departments.

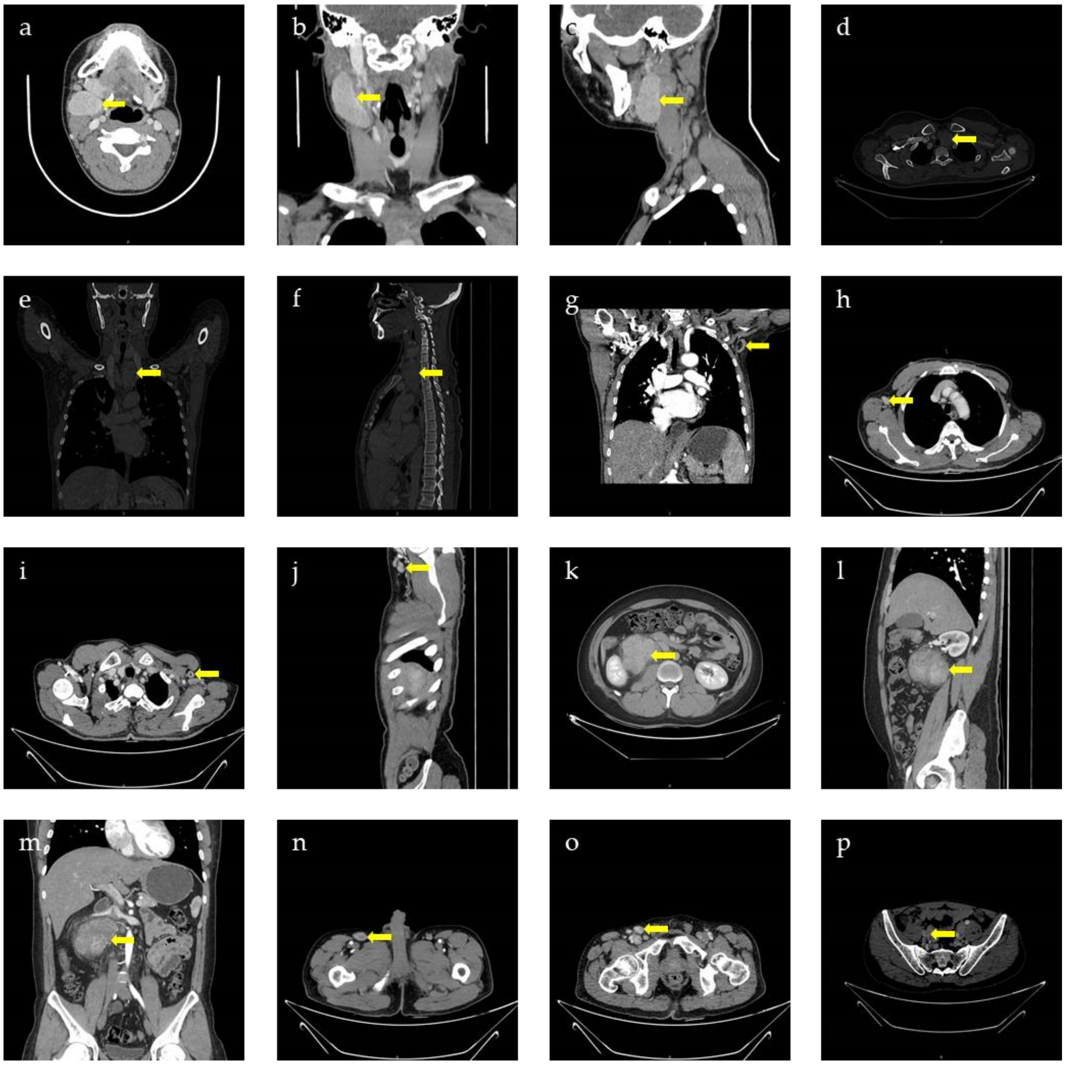


**Supplementary Figure S3. Radiological examinations.** CT images on different planes showed the enlarged lymph nodes in neck (a-c), mediastinum (d-f), axilla (g-j), abdomen (k-m), groin (n-o) and pelvis (p) of 6 patients with CD, among which patients with lymphadenopathies in neck/mediastinum/abdomen were with UCD/HV subtype and the other three were with MCD/PC subtype.

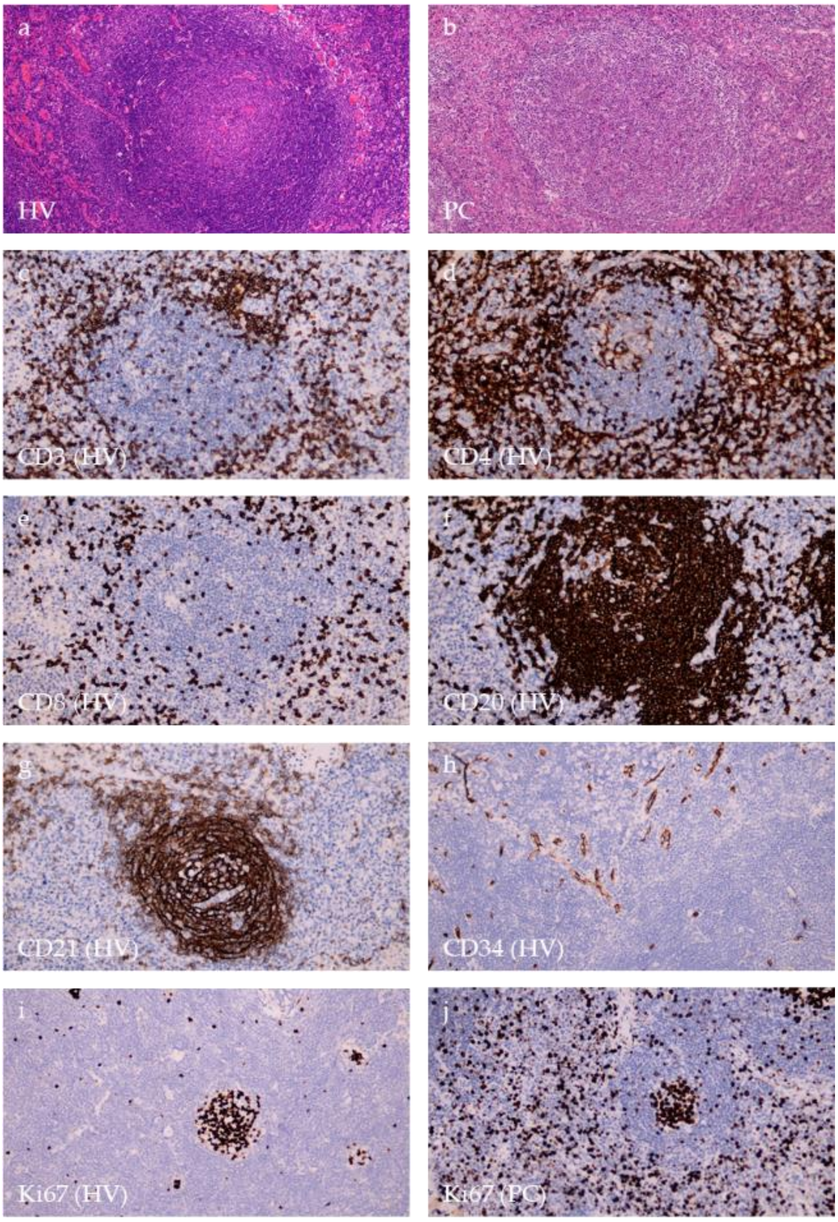


**Supplementary Figure S4. HE and IHC staining.** a. HE staining of an HV variant (×100); b. HE staining of a PC variant (×100); c. CD3-IHC staining of an HV variant (×200); d. CD4-IHC staining of an HV variant (×200); e. CD8-IHC staining of an HV variant (×200); f. CD20-IHC staining of an HV variant (×200); g. CD21-IHC staining of an HV variant (×100); h. CD34-IHC staining of an HV variant (×100); i. Ki67-IHC staining of an HV variant (×100); j. Ki67-IHC staining of a PC variant (×100).

| Index | Count | Index | Count | Index | Count | Index | Count |
|---|---|---|---|---|---|---|---|
| Ki67 | 65 | HHV-8 | 15 | Factor-8 | 4 | CK8 | 1 |
| CD20 | 63 | IgG | 14 | AFB | 4 | c-Myc | 1 |
| CD3 | 62 | IgG4 | 14 | TB-DNA | 4 | F XIII A | 1 |
| CD21 | 54 | CD79a | 13 | CD99 | 3 | Granzyme B | 1 |
| Pax-5 | 49 | CD35 | 12 | CXCL-13 | 3 | Hepatocyte | 1 |
| CD23 | 42 | CD4 | 11 | Desmin | 3 | MLH1 | 1 |
| CD10 | 41 | CD163 | 11 | LCA | 3 | MSH2 | 1 |
| EBER | 41 | CK (AE1/AE3) | 11 | P63 | 3 | MSH6 | 1 |
| CD138 | 37 | CD68 | 9 | ALK | 2 | Mucin-1 | 1 |
| Bcl-2 | 37 | CD15 | 8 | CD7 | 2 | Oct-2 | 1 |
| CD38 | 35 | EMA | 8 | CD117 | 2 | P53 | 1 |
| CD5 | 34 | CD1a | 7 | CK20 | 2 | PD-1 | 1 |
| Mum-1 | 34 | CD8 | 7 | SMA | 2 | PD-L1 (22C3) | 1 |
| CD43 | 30 | CD123 | 7 | TIA-1 | 2 | PMS2 | 1 |
| Bcl-6 | 27 | TdT | 7 | IgM | 2 | Villin | 1 |
| Kappa | 27 | S-100 | 6 | Arginase-1 | 1 | HBcAg | 1 |
| Lambda | 27 | CD31 | 5 | Bob-1 | 1 | HBsAg | 1 |
| CD34 | 22 | CD56 | 5 | CgA | 1 | IGH/IGK gene rearrangement | 1 |
| Cyclin-D1 | 19 | CK | 4 | CK/D2-40 | 1 | PAS | 1 |
| CD2 | 16 | CK19 | 4 | CK5/6 | 1 | TCR seq | 1 |
| CD30 | 16 | EBNA2 | 4 | CK7 | 1 | | |

**Supplementary Figure S5. Choices of pathological index for CD.** 83 pathological indexes (including IHC and molecular pathology) were listed in order of the number of times they were tested in 65 patients with CD in this study.

**Supplementary Table S1 Epidemiologic characteristics of patients with UCD and MCD**

| | **UCD** | **MCD** | **Total no. of patients with available data** |
|---|---|---|---|
| **Sex, n (%)** | 33 (50.8%) | 32 (49.2%) | 65 |
| Male | 13 (39.4%) | 21 (65.6%) | 34 (52.3%) |
| Female | 20 (60.6%) | 11 (34.4%) | 31 (47.7%) |
| **Pathological subtype, n (%)** | 33 (50.8%) | 32 (49.2%) | 65 |
| HV | 27 (81.8%) | 12 (37.5%) | 39 (60.0%) |
| PC | 3 (9.1%) | 19 (59.4%) | 22 (33.8%) |
| Mixed | 3 (9.1%) | 1 (3.1%) | 4 (6.2%) |
| **Age at disease onset (years), mean ± SD (min-max), n (%)** | 36.1 ± 18.0 (2-73), 32 (50.0%) | 40.9 ± 19.9 (3-71), 32 (50.0%) | 38.5 ± 18.9 (2-73), 64 |
| HV | 34.8 ± 17.7 (2-73), 26 (81.3%) | 35.8 ± 20.9 (5-66), 12 (37.5%) | 35.1 ± 18.5 (2-73), 38 (59.4%) |
| PC | 54.7 ± 5.5 (51-61), 3 (9.4%) | 44.0 ± 19.6 (3-71), 19 (59.4%) | 45.5 ± 18.6 (3-71), 22 (34.4%) |
| Mixed | 28.7 ± 21.2 (6-48), 3 (9.4%) | 43, 1 (3.1%) | 32.3 ± 18.7 (6-48), 4 (6.3%) |
| **Diagnosis delay (months), median, n (%)** | 1.25, 32 (50.0%) | 3.00, 32 (50.0%) | 2.50, 64 |
| HV | 0.79, 26 (81.3%) | 3.50, 12 (37.5%) | 2.00, 38 (59.4%) |
| PC | 1.50, 3 (9.4%) | 2.50, 19 (59.4%) | 2.50, 22 (34.4%) |
| Mixed | 3.00, 3 (9.4%) | 0.46, 1 (3.1%) | 2.00, 4 (6.3%) |

**Supplementary Table S2 Clinical findings of patients with UCD and MCD**

| | UCD | MCD | Total no. of patients with available data |
|---|---|---|---|
| **Location of lymphadenopathy, n (%)** | 33 (50.8%) | 32 (49.2%) | 65 |
| Neck | 10 (30.3%) | 21 (65.6%) | 31 (47.7%) |
| Abdomen | 13 (39.4%) | 6 (18.8%) | 19 (29.2%) |
| Axilla | 0 | 8 (25.0%) | 8 (12.3%) |
| Groin | 1 (3.0%) | 7 (21.9%) | 8 (12.3%) |
| Mediastinum | 4 (12.1%) | 3 (9.4%) | 7 (10.8%) |
| Pelvis | 3 (9.1%) | 2 (6.3%) | 5 (7.7%) |
| Parotid gland | 1 (3.0%) | 1 (3.1%) | 2 (3.1%) |
| Lung | 1 (3.0%) | 0 | 1 (1.5%) |
| Elbow | 1 (3.0%) | 0 | 1 (1.5%) |
| **Signs and symptoms, n (%)** | 33 (50.8%) | 32 (49.2%) | 65 |
| Fever | 0 | 10 (31.3%) | 10 (15.4%) |
| Cough | 0 | 2 (6.3%) | 2 (3.1%) |
| Marasmus | 0 | 1 (3.1%) | 1 (1.5%) |
| **Comorbidities, n (%)** | 9 (27.3%) of 33 | 10 (31.3%) of 32 | 19 (29.2%) of 65 |
| Liver | 5 (15.2%) | 3 (9.4%) | 8 (12.3%) |
| Splenomegaly | 2 (6.1%) | 4 (12.5%) | 6 (9.2%) |
| Oedema/effusion | 1 (3.0%) | 3 (9.4%) | 4 (6.2%) |
| Kidney | 1 (3.0%) | 3 (9.4%) | 4 (6.2%) |
| Lung | 2 (6.1%) | 1 (3.1%) | 3 (4.6%) |
| Thyroid | 2 (6.1%) | 1 (3.1%) | 3 (4.6%) |
| Gallbladder | 0 | 1 (3.1%) | 1 (1.5%) |
| Stomach | 1 (3.0%) | 0 | 1 (1.5%) |
| Ovary | 1 (3.0%) | 0 | 1 (1.5%) |
| Femur | 1 (3.0%) | 0 | 1 (1.5%) |
| Paroxysmal disturbance of consciousness | 0 | 1 (3.1%) | 1 (1.5%) |

**Supplementary Table S3 Pathological findings of patients with UCD and MCD**

| | UCD | MCD | Total no. of patients with available data |
|---|---|---|---|
| **HHV-8, n (%)** | 8 | 7 | 15 |
| Positive | 0 | 1 (14.3%) | 1 (6.7%) |
| Negative | 8 (100%) | 6 (85.7%) | 14 (93.3%) |
| **Major diameter of pathological specimen (cm), mean ± SD (min-max), n (%)** | 6.4 ± 2.2 (2.5-12), 33 (50.8%) | 3.1 ± 1.9 (0.6-8.0), 32 (49.2%) | 4.7 ± 2.6 (0.6-12.0), 65 |
| HV | 6.5 ± 2.2 (3.0-12.0), 27 (81.8%) | 3.5 ± 1.9 (1.0-6.6), 12 (37.5%) | 5.6 ± 2.5 (1.0-12.0), 39 (60.0%) |
| PC | 7.3 ± 1.5 (6.0-9.0), 3 (9.1%) | 2.9 ± 2.0 (0.6-8.0), 19 (59.4%) | 3.5 ± 2.5 (0.6-9.0), 22 (33.8%) |
| Mixed | 4.3 ± 1.8 (2.5-6.0), 3 (9.1%) | 1.2, 1 (3.1%) | 3.6 ± 2.1 (1.2-6.0), 4 (6.2%) |

**Supplementary Table S4 Chemotherapy regimens and follow-up**

| | Patient no. | Chemotherapy regimens | Follow-up time (months) | Follow-up results |
|---|---|---|---|---|
| UCD | 1 | lansoprazole + vidarabine monophosphate + lienal polypeptide | 80 | alive, NR |
| | 2 | no detail | 90 | loss of follow up |
| MCD | 3 | CHOP | 32 | alive, NR |
| | 4 | CHOP | 23 | died, PD |
| | 5 | CHOP | 105 | alive, NR |
| | 6 | CHOP | 13 | alive, NR |
| | 7 | CHOP | 101 | alive, NR |
| | 8 | CHOP | 88 | loss of follow up |
| | 9 | CHOP | 84 | died, PD |
| | 10 | CHOP + CHOPE | 98 | alive, NR |
| | 11 | CHOP + IFN-α + thalidomide | 101 | alive, NR |
| | 12 | VD + VAD + CHOP + CHOPE | 105 | alive, NR |
| | 13 | TCP | 18 | loss of follow up |
| | 14 | pirarubicin (THP) + prednisone + vincristine sulfate (Oncovin) + tropisetron | 81 | alive, NR |
| | 15 | rituximab + methylprednisolone | 16 | alive, NR |
| | 16 | rituximab | 35 | alive, NR |
| | 17 | no detail | 27 | alive, NR |
| | 18 | no detail | 66 | alive, NR |

CHOP: cyclophosphamide + doxorubicin hydrochloride (hydroxydaunorubicin) + vincristine sulfate (Oncovin) + prednisone; CHOPE: CHOP + etoposide phosphate; VD: bortezomib (Velcade) + dexamethasone; VAD: vincristine + doxorubicin (Adriamycin) + dexamethasone; TCP: thalidomide + cyclophosphamide + prednisolone; NR: no recurrence; PD: progressive disease
